# Infection-Associated Flares in Systemic Lupus Erythematosus

**DOI:** 10.3390/pathogens13110934

**Published:** 2024-10-26

**Authors:** Giuseppe A. Ramirez, Chiara Calabrese, Marta Secci, Luca Moroni, Gabriele D. Gallina, Giovanni Benanti, Enrica P. Bozzolo, Marco Matucci-Cerinic, Lorenzo Dagna

**Affiliations:** 1 Unit of Immunology, Rheumatology, Allergy and Rare Diseases, IRCCS Ospedale San Raffaele, Via Olgettina 60, 20132 Milan, Italy; calabrese.chiara@hsr.it (C.C.); secci.marta@hsr.it (M.S.); moroni.luca@hsr.it (L.M.); gallina.gabriele@hsr.it (G.D.G.); benanti.giovanni@hsr.it (G.B.); bozzolo.enrica@hsr.it (E.P.B.); matuccicerinic.marco@hsr.it (M.M.-C.); dagna.lorenzo@hsr.it (L.D.); 2 Faculty of Medicine, Università Vita-Salute San Raffaele, Via Olgettina 58, 20132 Milan, Italy; 3 Faculty of Medicine, Università degli Studi di Cagliari, Strada Provinciale 8, 09042 Monserrato (CA), Italy

**Keywords:** lupus, infections, flare, herpesvirus, LLDAS, remission

## Abstract

Systemic lupus erythematosus (SLE) is characterised by generalised immune dysfunction, including infection susceptibility. Infection-associated flares (IAFs) are common and might rapidly self-resolve, paralleling infection resolution, but their specific clinical phenotype is poorly understood. Therefore, we screened 2039 consecutive visits and identified 134 flares, defined as a loss of the lupus low disease activity state (LLDAS), from 1089 visits at risk spanning over multiple follow-up years, yielding an average yearly LLDAS deterioration rate of 17%. Thirty-eight IAFs were isolated from the total flares and were mostly related to bacterial and herpesvirus infections. When compared to other flares (OFs; n = 98), IAFs showed no milder patterns of organ involvement and similar rates of long-term damage accrual, as estimated by conventional clinimetrics. Arthritis in IAFs was more severe than that in OFs [median (interquartile range) DAS-28 2.6 (2.3–4.1) vs. 2.0 (1.6–2.7); *p* = 0.02]. Viral IAFs were characterised by atypically lower levels of anti-DNA antibodies (*p* < 0.001) and possibly abnormally high complement levels when compared to flares of different origin. These data suggest that IAFs are of comparable or even higher severity than OFs and may subtend distinct pathophysiological mechanisms that are poorly tackled by current treatments. Further research is needed to confirm these data.

## 1. Introduction

Systemic lupus erythematosus (SLE) is a multi-organ disease with a wide spectrum of clinical presentations, sustained by generalised immune dysfunction [1,2]. In recent years, mechanistic and clinical studies have significantly improved our understanding of the pathophysiology of the disease, paving the way to innovative treatment strategies. The aberrant processing of self-antigens, favoured by permissive human leukocyte antigen repertoires and unsolicited antiviral-like interferon (IFN)-driven mechanisms, has been identified as the core pathogenic event, leading to non-physiological antibody responses and eventual organ damage [3,4,5,6,7,8]. Treatments aiming down- and up-stream of these events have progressively been introduced into clinical practice [9,10,11,12,13]. Nonetheless, patients with SLE still face a significant risk of morbidity and early mortality due to chronic and acute events secondary to disease activity and drug-related side-effects [14,15]. With infections, cardiovascular complications and organ failure due to active disease being the most significant causes of mortality [16], disease exacerbations (or *flares*) constitute a major driver of organ damage and a prominent therapeutic target for secondary prevention [15,17,18,19,20].

Prolonged remission has progressively emerged as a key clinical predictor of relapse-free survival in the long term [20,21], prompting the definition of accurate criteria to establish its attainment after treatment. In the setting of clinimetrics certifying remission, attainment of the Definitions Of Remission In SLE (DORIS) parameters [22] is currently regarded as the ideal target in the treatment of SLE. The lupus low disease activity state (LLDAS) [23] identifies a slightly broader set of patients with SLE enjoying a condition of limited active inflammation and relatively low treatment burden. Thanks to these characteristics, it may be more sensitive than the DORIS definition in intercepting a significant loss of disease stability in a wider fraction of patients with SLE, including those who have not achieved a complete remission state. Accordingly, it has been successfully validated to assess the occurrence of flares and predict damage accrual [24,25].

Beyond the generic association between failure to achieve remission and the risk of eventual flares, limited information is available about the determinants of lupus exacerbations and about potentially distinct flare profiles. Rising levels of anti-DNA antibodies (ADNAs) are generally associated with disease activity and may herald an incumbent flare in at least a fraction of patients with SLE [20,26]. However, other clinical and serological predictors may also correlate with eventual disease exacerbations, suggesting the existence of multiple pathogenic ways that can lead to the same detrimental phenotypic outcome [27,28,29].

Besides being immediate determinants of morbidity and a potential cause of death in patients with SLE [30,31], infections are also associated with an enhanced risk of disease exacerbation [32]. On the other hand, incomplete disease control and/or suboptimal treatment are also associated with a higher infectious risk in patients with SLE [32,33,34]. In fact, patients with SLE are charged with a disproportionately high susceptibility to infections [35,36], especially those due to airborne pathogens, including severe acute respiratory syndrome coronavirus 2 (SARS-CoV-2) [32,36,37,38]. Influenzavirus is a major driver of disease flares due to its widespread diffusion [32]. Parvovirus B19 infection can also cause disease exacerbation, besides constituting a known mimicker of SLE [39,40]. In addition, the reactivation of latent viral agents, including Epstein–Barr virus (EBV), cytomegalovirus (CMV), Varicella Zoster Virus (VZV) and other Herpesviridae, along with endogenous retroviral elements, is also a pathogenic hallmark of SLE [41,42,43]. In particular, EBV has emerged as a major trigger of autoimmune phenomena within and beyond SLE, possibly also due to the molecular mimicry between key autoantigens and EBV components [44,45,46]. All these pathogens might boost SLE-related aberrations in the immune response in multiple ways, including para-physiological stimulation of innate immune mechanisms (such as IFN pathways), molecular mimicry with key endogenous antigens [45] and heterologous T-cell immunity [8,47,48]. Vaccination represents the cornerstone of prevention for patients with SLE. Most local and international guidelines recommend active immunisation of patients with SLE against a broad set of viral and bacterial pathogens in addition to standard vaccinations. Specifically, seasonal viral pathogens (such as influenza and, in more recent times, possibly SARS-CoV-2), along with pneumococcus and human papillomavirus, constitute the core target of vaccination in patients with SLE [49,50]. VZV immunisation with the recent recombinant adjuvanted vaccine is also part of the international recommendations for patients with SLE [50]. Extended immunisation against other capsulate bacteria such as meningococcus and *Haemophilus* is usually recommended in case of immune suppression, especially with B-cell-depleting strategies [51]. Although the response rates to vaccination can be lower in patients with SLE, due to disease-related immune dysfunction and/or immune suppression, safety and efficacy data support their extensive use to prevent clinically relevant complications [52,53,54,55,56,57]. Nonetheless, vaccination coverage is often disappointingly lower than the targets set by international guidelines [58,59,60]. This contributes to infection susceptibility and eventually to flare proneness in patients with SLE.

Little is known about potential clinical divergences between SLE-related events associated with infectious triggers and fluctuations in disease activity putatively related to other factors. Specifically, very limited information is available on the clinical characteristics of infection-associated flares (IAFs) in comparison with other SLE flares (OFs).

To address this issue, we set up an observational study on a relatively large cohort of patients with SLE longitudinally followed up with the aim of evaluating potential clinical and laboratory differences between IAFs and OFs.

## 2. Materials and Methods

### 2.1. Patient and Visit Selection

**Figure 1:**
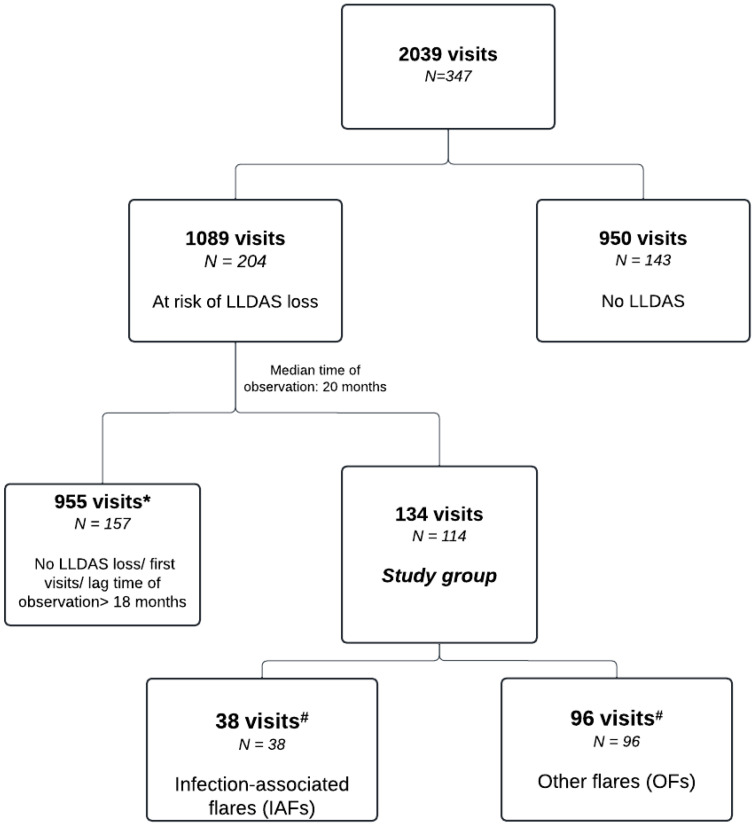
Study flowchart. *: patients with visits fitting the study criteria were included into this and the “study group”, #: 20 patients had both IAF and OF records.

We retrospectively analysed a set of prospectively collected data from 347 patients with SLE who fulfilled the 2019 EULAR/ACR Classification Criteria for Systemic Lupus Erythematous [61] and, upon giving their written informed consent, were consecutively enrolled in the PanImmuno Research Protocol, which conformed to the Declaration of Helsinki and was approved by San Raffaele Institutional Review Board (approval number 22/INT/2018). Specifically, all consecutive visits recorded in the period between July 2015 and December 2023 at San Raffaele University Hospital Lupus Clinic were scrutinised. Longitudinal data were collected through dedicated in-house software built in a Microsoft Excel^®^ 2019 environment [25]. Among the whole set of visits (Figure 1), we identified visit couples consisting of a visit performed at the time of flare and a pre-flare visit, setting the maximum lag time between the two visits at 18 months.

We defined a disease flare as a loss of the LLDAS [23] across two consecutive outpatient visits. First visits (patients at disease onset or newly referred to the Centre) were excluded. We then dichotomised flares into two groups: flares associated with a recent infection (IAFs), defined as an infectious event occurring between visits and requiring antimicrobial treatment and/or absence from work/school, and flares without any clear evidence of recent infection (OFs). Infections were attributed to a bacterial aetiology in case of proper isolation of non-contaminating bacteria in culture tests, the involvement of typical sites such as the urinary tract, characteristic clinical presentations (such as respiratory tract infections with purulent secretions), increased acute-phase reactants including C-reactive protein (CRP), and/or a response to antibiotics after no response to other treatments. Viral infections were defined as events without the abovementioned bacterial-like features and without evidence of active SLE and/or with serological or genomic evidence of active viral replication. Fungal infections were defined by the presence of typical yeast-related lesions or secretions in typical mycotic sites or positive culture tests. No data were homogeneously available in terms of antimicrobial treatments, since the treatment of infectious complications is usually managed in primary care. Immunosuppressant discontinuation during antimicrobial treatment was advised to all patients.

### 2.2. Clinical and Laboratory Data

The collected clinical data encompassed demographics, past and current SLE-related manifestations, comorbidities and treatments. SLE severity was measured by using the SLE Disease Activity Index 2000 (SLEDAI-2K) [45] (in terms of the absolute score and variation in the score (delta) across pre-flare and flare visits), the British Isles Lupus Assessment Group (BILAG) 2004 index [46] and a 0.0–3.0 physician global assessment scale (PGA) [62]. Patients’ impressions about their global health status were also quantitated through a 0–10 numerical rating scale (NRS), with 10 corresponding to the highest perceived degree of wellbeing. Chronic damage was measured by employing the SLE International Collaborating Clinics/American College of Rheumatology Damage index (SDI) [47]. Joint disease activity was estimated with the DAS-28 score.

We also collected data on patient vaccination status at the time of flare. Since the recent COVID-19 pandemic has caused major perturbations in health policies, including the introduction of a new set of vaccines, we categorised study flares into flares occurring before or after the pandemic. We considered 1 January 2020 as the starting date of the pandemic.

We used laboratory data acquired in the framework of standard clinical practice (CRP, erythrocyte sedimentation rate—ESR, creatinine, 24 h proteinuria). Due to the high variability among laboratories, complement consumption was coded dichotomously into low vs. normal C3 and/or C4. Anti-double-stranded DNA antibody (ADNA) titres were classified using a 0–4 discrete arbitrary scale to homogenise the results from different laboratory ranges of normality.

### 2.3. Statistical Analyses

We analysed the intra- and interindividual changes accompanying disease flares among the groups. The data for descriptive statistics are expressed as the median (interquartile range) or percentage unless otherwise specified. Continuous variables were tested for normality by using the Shapiro–Wilk test. Non-normally distributed continuous variables were compared between two groups by using the Mann–Whitney U-test. Intraindividual comparisons were performed by using the Wilcoxon matched-pairs signed rank test. Associations between categorical variables were assessed by performing Chi-square tests. Time-dependent differential outcomes among groups were assessed through Cox regression analysis. All data are presented as the median (interquartile range, IQR) or percentage unless otherwise specified. Data that are presented as stratified results among groups were all tested to identify statistically significant differences. *p*-values of <0.05 were considered significant and reported, unless otherwise specified. Data were analysed with JASP version 0.19.0 and Statacorp STATA version 15.0.

## 3. Results

### 3.1. Demographics and General Clinical Characteristics

Out of 2039 consecutive visits by 347 patients, we identified 1089 visits by patients with previous LLDAS attainment and at risk for deterioration. Over a median time of observation of 20 (9–38) months, 134/1089 (12%) visits from 114 patients met the criteria for an IAF (n = 38 flares) or OF (n = 96 flares; Figure 1). Eighty-four flares occurred before the onset of the COVID-19 pandemic: 35% (n = 29) were IAFs and 65% (n = 55) were OFs.

The average yearly loss rate of the LLDAS was 17% among patients at risk, with higher flare rates during the first years of observations (Supplementary Materials Table S1). Most patients with available visits were women (n = 100/114). The median age of the cohort was 44 (35–52) years. The median follow-up time at the index flare was 30 (13–62) months. The median lag time between the pre-flare and flare visits was 6 (4–8) months. The most frequent clinical manifestations in the patients’ history involved the haematological (82%), mucocutaneous (81%) and musculoskeletal (80%) domains. Eighty-five patients had a history of positive ADNA (79% of IAF patients and 74% of OF patients), while sixty-two patients had at least one positive antiphospholipid antibody in their history (Table 1).

### 3.2. Pre-Flare Disease Status

At the time of the last LLDAS before a flare, the patient NRS was 7 (6–8). Accordingly, the total BILAG score was 1 (0–1), indicating an absence of/low disease activity. Modest alterations were only found in the haematological and mucocutaneous domains. In all, 109 patients (31/38 in the IAF group and 78/96 in the OF group) had serological activity at pre-flare. The differences between the two groups were not statistically significant (Table 2). At the pre-flare visit, 82 patients were under at least one immunosuppressant (42% of IAFs, 69% of OFs), while 120/134 were taking hydroxychloroquine (82% of all IAFs and 84% of all OFs). Due to persistent disease stability, corticosteroids were tapered in 21 cases (11/16 patients on steroids in the IAF group and 10/41 in the OF group; *p* = 0.005) and discontinued in 13 cases (4/16 IAFs and 9/41 OFs; *p* > 0.999) at the end of the visit. No patient discontinued immunosuppressants.

### 3.3. Flare Profiles by Groups

#### 3.3.1. Characteristics of Infection-Associated Flares

**Figure 2:**
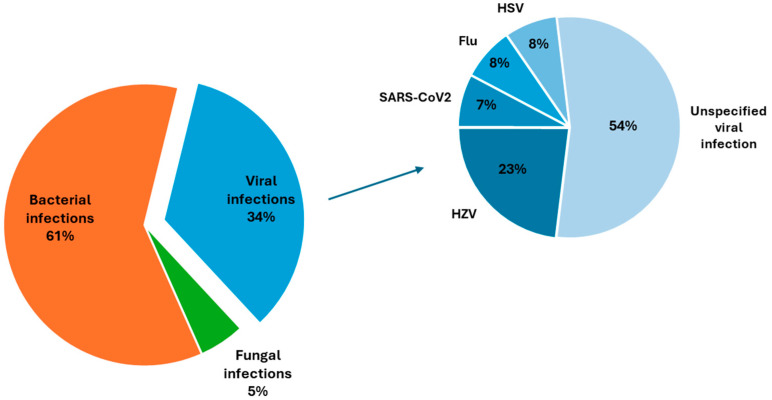
Pie charts showing the differential pathogenic distribution of infections in IAF patients (**left**) and in detail in the viral subgroup (**right**).

Putative bacterial infections were linked to 23/38 (61%) IAF cases. Thirteen cases (34%) were associated with a viral infection/reactivation. Two flares (5%) followed a fungal infection. Among the bacterial IAFs, 13 were associated with a respiratory tract infection, including 1 case of pneumonia and 12 cases of upper airway infections. Eight patients had a bacterial urinary tract infection preceding an IAF. One case of bacterial conjunctivitis and one case of gastroenteritis were also recorded. In the viral subgroup, Herpesviridae were responsible of four events (30%). Three cases were attributed to Varicella Zoster Virus (VZV) and one case to Herpes Simplex Virus (HSV). One case of viral IAF was associated with a recent severe acute respiratory syndrome coronavirus 2 (SARS-CoV-2) infection. Eight cases were classified as flu-like respiratory syndrome of potential viral aetiology, but no specific data were available about the insulting agent. In the IAF cohort, none of the patients with Herpes Zoster infection/reactivation were under steroids, although two out of four were on immunosuppressants. Both cases of fungal infection were sustained by *Candida* spp. (Figure 2).

At the time of flare (for both IAFs and OFs), the vaccine coverage rates were 68% (n = 91) for the anti-influenza vaccine, 59% (n = 79) for the anti-pneumococcal disease vaccine, 16% (n = 22) for the tetravalent meningococcal vaccine, 11% (n = 15) for the B meningococcal vaccine, 5% (n = 7) for the anti-Haemophilus influenzae vaccine and 11% (n = 15) for the recombinant HZV disease vaccine. Fifty flares (nine IAFs and forty-one OFs) occurred during the COVID-19 pandemic. Twenty-eight of them occurred when anti-COVID-19 vaccines were available. Twenty patients out of these twenty-eight (71%) were vaccinated at the time of flare. No statistical differences were found when the vaccination profiles of IAF and OF patients at the time of flare were compared.

#### 3.3.2. Clinical and Laboratory Features

**Figure 3:**
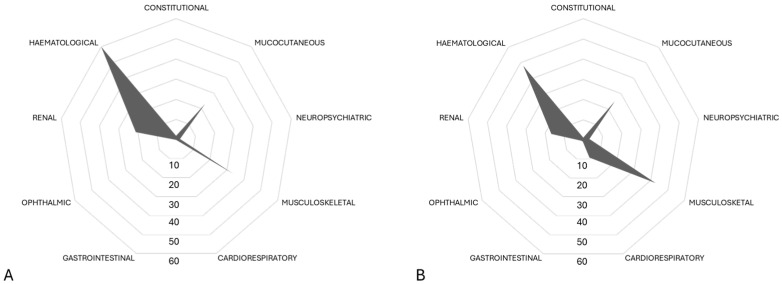
(**A**,**B**); Radar plots showing the differential distribution of active BILAG domains among patients with OFs (**A**) and IAFs (**B**). Data are expressed as percentages. In both cases, the most represented BILAG domains were the haematological, musculoskeletal and mucocutaneus domains. BILAG: British Isles Lupus Assessment Group.

The differential clinical profile of patients with IAFs and OFs was evaluated by comparing the number and type of active BILAG domains. In patients with IAFs, one or two BILAG domains were active in 80% of cases. Haematological (60%), musculoskeletal (34%), mucocutaneous (23%) and renal (21%) domains were most frequently involved. Cardiopulmonary manifestations were numerically less frequent in IAFs than OFs (0/38 vs. 9/96; *p* = 0.060; Figure 3). No other differences were observed with regard to active BILAG domains among the OF and IAF groups. Viral and bacterial IAFs also showed similar patterns of BILAG domain involvement at the time of flare.

In terms of biochemical parameters at the time of flare, there were no statistically significant differences in the frequency of low C3 or C4 complement levels or in ADNA titres when patients with all types of IAF and patients with OFs were compared. However, positive ADNA was represented more among patients with bacterial IAFs (17/23) than in patients with viral IAFs (5/13; *p* = 0.036). Consistently, patients with bacterial IAFs more frequently had a history of positive ADNA than did patients with viral IAFs (96% vs. 46%; *p* < 0.001). Low complement was numerically more frequent among bacterial IAFs than among viral IAFs (13/23 vs. 3/13, respectively; *p* = 0.052). Similar trends were observed when viral IAFs were compared to OFs. No differences were found when biochemical parameters of the bacterial subgroup and OFs were compared. Platelet counts were relatively lower in patients with viral IAFs than in patients with OFs (*p* = 0.037), although the majority of observations fell within the range of normality. In addition, no significant differences were found in comparisons of the clinical features or biochemical profiles of OFs and IAFs stratified by onset before vs. after the COVID-19 pandemic. All clinical and laboratory features of the patients are summarised in Table 3.

#### 3.3.3. Flare Severity

Flare severity was studied by analysing the SLEDAI-2K absolute scores at the time of flare and variations (delta) between pre-flare and flare visits. There were no significant differences between IAFs and OFs in terms of absolute SLEDAI-2K. The degree of SLEDAI-2K deterioration from pre-flare to flare status was also comparable among the groups. The total BILAG and PGA scores did not differ between OFs and IAFs, nor among IAF subgroups. The patient-reported NRS was also comparable among the groups. Despite a comparable frequency of musculoskeletal manifestations among patients with IAFs and OFs, higher DAS-28 scores [2.6 (2.3–4.1), n = 12] were found in IAFs than in OFs [2.0 (1.6–2.7), n = 47; *p* = 0.024]. Accordingly, when DAS-28 scores at the time of flare were compared intraindividually among patients who experienced both an IAF and an OF, higher DAS-28 scores were found during IAFs than during OFs (10/11 vs. 1/11; *p* = 0.004). The flare treatment strategies were similar among the groups.

### 3.4. Long-Term Disease Course

**Figure 4:**
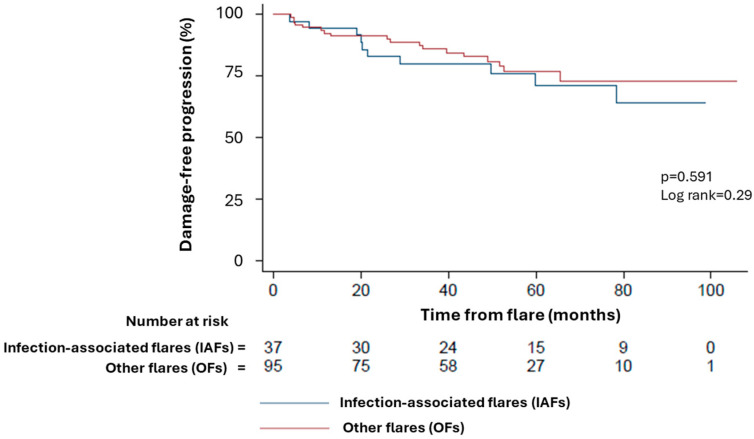
Kaplan–Meier survival curves showing the rate of damage accrual in patients with IAFs (blue line) and OFs (red line) over time.

Patients experiencing IAFs (whether of viral, bacterial or fungal origin) had a similar likelihood of chronic damage progression over time when compared to those experiencing OFs (Log-rank = 0.29; hazard ratio 0.80, 95% CI: 0.37–1.75; *p* = 0.591; Figure 4). Furthermore, the monthly SDI item accrual rates were also comparable among OFs (0 (0–1)/100 months), IAFs (0 (0–0) items/100 months) and IAF subgroups.

## 4. Discussion

In this study, we found that patients with SLE in an LLDAS are exposed to a 17% annual risk of clinical deterioration, with more than one-quarter of flare cases being associated with a recent infection, most frequently of bacterial origin and affecting the upper respiratory tract. While IAFs and OFs were both characterised by prominent haematological and musculoskeletal manifestations, IAFs presented with arthritis of higher severity and showed converging damage accrual trends in the long term when compared to OFs.

Despite the availability of novel treatments and of more rational uses of existing therapeutic weapons, paving the way to ambitious targets, disease relapses are still common in the majority of patients with SLE. Consistent with our observations, previous studies have shown that more than 10% of patients with SLE lose a previously attained LLDAS each year [63]. This phenomenon tends to attenuate at later stages of the disease and be more prominent soon after LLDAS attainment [64] or closer to disease onset [65], with no clear risk-free conditions other than persistent LLDAS itself [20]. Infections have long been regarded as a major cause of morbidity (and potentially of mortality) in patients with SLE. Ever-growing evidence also supports a role of infections in favouring disease exacerbations. In contrast to the characteristic self-resolving course of SLE-related symptoms following microbial antigen stimulation in the setting of vaccines [66,67], wild-type infections in SLE are typically more severe, of longer course and associated with full-blown disease flares [32,68,69,70], possibly indicating that SLE pathogenic events potentially triggered by infections are unlikely to self-resolve and might be more resistant to treatments aiming at classical aseptic inflammatory mechanisms [71]. In line with this argument, intraindividual (besides interindividual) comparisons of arthritis severity at the time of flares in our cohort revealed a more severe joint involvement following infections.

In line with previous reports [33,36,38,72], bacterial infections were more frequent than viral infections in patients classified within the IAF group and were mainly localised to the respiratory tract. Inborn or acquired deficits of the complement system coexist in patients with SLE and contribute to the hallmark finding of low serum complement levels in patients with active disease [73]. In addition, they may concur with SLE-related susceptibility to bacterial infections [70,71]. Accordingly, a trend towards lower levels of complement was observed in patients with bacterial IAFs in this study.

Patients with viral IAFs constituted a standalone subgroup in the context of IAFs and were characterised by a lower frequency of typical SLE serological alterations (that is, positive ADNA and possibly low complement). The most frequent viral IAFs were sustained by VZV and other herpesviruses and occurred in patients who were off corticosteroids, in line with SLE’s intrinsic susceptibility to herpesviruses [41]. Regarding this, recent works have also shown that a fraction of patients with SLE might harbour natural anti-IFN neutralising antibodies among their serological repertoire. These antibodies might interfere with IFN-dependent mechanisms of inflammation, reducing the likelihood of SLE flares at the price of a higher susceptibility to infections [74,75]. Adding to previous evidence, we found that patients susceptible to disease flares after infection/viral reactivation have, however, no milder clinical presentation when compared to patients with flares without clear associations with infectious triggers, and they accrue similar amounts of chronic damage in the long term. Taken together, these data further support the hypothesis of unconventional mechanisms of inflammation occurring in IAFs in contrast to OFs, which might only be partially tackled by standard treatment approaches, currently chosen according to the clinical phenotype only. Ultimately, this can have a detrimental impact on patient prognosis and possibly account for the significant burden of damage accrual observed in patients with IAFs, similar to that in OFs. Consistent with this hypothesis, recent studies indicate an association between more complex alterations of multiple branches of the immune response and enhanced flare risk in patients with SLE [76].

Another potential consequence of the existence of atypical disease mechanisms in all or some patients with higher susceptibility to infections and to IAFs might be that some clinical manifestations of morbidity in these patients are not correctly captured by current clinimetrics. Indeed, the recent severe acute respiratory syndrome coronavirus 2 health crisis has highlighted the detrimental impact of difficult-to-diagnose/-treat post-infectious long-term syndromes on patient quality of life [77]. Consistent with this, post-infection-like symptoms such as fatigue, chronic pain or low-grade mood disorders are well-known features of SLE and major causes of disability, despite their relatively low weight in current SLE clinimetrics [78,79,80,81,82,83]. Patients with IAFs might therefore only apparently present with a comparable burden of disease compared to OFs due to the limitations of current clinical assessment tools. Unfortunately, the data from our research are insufficient to test this challenging hypothesis, warranting the acquisition of further data from ad hoc studies. The potential existence of a clinical/pathophysiological discrepancy between patients with IAFs and other SLE patients might, however, have clinical implications. For example, disease stability might be overestimated in patients at risk for IAFs. Consistent with this, steroid tapering but not final discontinuation pre-flare was associated with eventual IAFs, possibly indicating that enhanced monitoring during corticosteroid tapering might be advised in patients with atypical SLE features [84,85,86]. Vaccination constitutes the mainstay of prevention in patients with SLE and might constitute the key solution to uncoupling enhanced disease instability due to treatment tapering and infectious risk. Notably, despite being consistent with data from the literature, the vaccine coverage rates in our cohort were disappointingly lower than those indicated by local and international guidelines [58,59,60]. This evidence highlights the importance of vaccination and patient education on this topic as a major modifiable unmet need for patients with SLE, especially in the context of growing vaccine hesitancy due to mistrust in public institutions and science [87,88]. In line with this view, the impact of the COVID-19 pandemic in patients with SLE (in terms of both infections and disease flares) was proportional to the extent of public health measures, including vaccination [56,89].

Additional studies are required to overcome other potential areas of uncertainty unaddressed by this study due to its limitations. In particular, due to the absence of data on variations in biohumoral parameters among the pre-flare, post-infection and overt flare status, our mechanistic hypotheses to interpret the study clinical findings remain speculative. Along the same lines, insufficient direct microbiological evidence of pre-flare infections might have introduced potential biases in classifying IAF patients. Furthermore, as a large fraction of infectious events were managed in primary care, we were not able to take into account potential biases related to the types of antimicrobial treatment and concomitant medications in patients eventually developing IAFs. Despite the relatively significant number of patients in the starting cohort, the number of analysed subjects and events was also quite low, preventing an accurate dissection of less frequent features potentially differentiating IAFs and OFs. Our retrospective design also contributes to this limitation.

Notwithstanding the impact of these considerations on the strength of our findings, this study potentially opens new perspectives on a lesser-known aspect of SLE clinical/pathophysiological heterogeneity, that is, disease exacerbation in the setting of combined autoimmunity and infection susceptibility. A deeper understanding of SLE dynamics in the subset of patients with these characteristics might improve patient characterisation and selection for treatment in the setting of both clinical trials and routine practice and improve current management strategies towards more personalised approaches.

## 5. Conclusions

In conclusion, this study confirms that patients with SLE are exposed to a persistent risk of relapse, which can be associated with recent infections in more than one-quarter of cases. SLE flares following a recent infection present with similar clinical manifestations compared to other disease exacerbations but might cause more severe arthritis and show atypical serological features in association with recent viral infections. Further studies are required to investigate the existence of potentially unconventional mechanisms sustaining SLE flares in infection-susceptible subjects and possibly design tailored treatment strategies to tackle them.

## Figures and Tables

**Table 1:** Demographic, comorbidity and SLE disease characteristics and therapies of patients.

	All Patients (n = 114)	OFs (n = 96)	IAFs (n = 38)
**Demographics**			
Women: n (%)	100 (88)	85 (88)	33 (87)
Age at disease onset (years): median (IQR)	28 (20–36)	29 (20–37)	26 (20–37)
Age at time of flare (years): median (IQR)	44 (35–52)	44 (35–52)	32 (34–54)
Follow-up duration at time of flare (months): median (IQR)	30 (13–62)	34 (15–71)	22(12–43)
**General clinical characteristics (history): n (%)**			
Musculoskeletal involvement	93 (80)	78 (81)	30 (79)
Mucocutaneous involvement	82 (81)	70 (72)	26 (68)
Renal involvement	47 (40)	38 (39)	17 (44)
NPSLE	28 (24)	22 (22)	10 (26)
Cardiopulmonary involvement	14 (12)	9 (9)	6 (15)
Haematological manifestations	94 (82)	78 (81)	30 (79)
Constitutional symptoms	46 (40)	38 (39)	17 (45)
Gastrointestinal manifestations	1 (1)	1 (1)	0 (0)
Anti-phospholipid syndrome	11 (10)	9 (9)	6 (16)
**Serology (history): n (%)**			
Anti-dsDNA	85 (74)	71 (74)	30 (79)
Anti-Sm	30 (26)	24 (25)	10 (26)
Low complement (C3 and/or C4)	31 (27)	27 (28)	11 (29)
Antiphospholipid antibodies			
▪ Anticardiolipin IgG or IgM	35 (30)	29 (30)	13 (34)
▪ Anti-Beta-2-glycoprotein I IgG or IgM	18 (16)	18 (18)	7 (18)
▪ LAC	23 (20)	20 (1)	8 (21)

NPSLE: neuropsychiatric systemic lupus erythematosus; LAC: lupus anticoagulant.

**Table 2:** Clinical and treatment features at the last visit before flare.

	All Flares (n = 134)	OFs (n = 96)	IAFs (n = 38)	Viral IAFs (n = 13)	Bacterial IAFs (n = 23)
**Disease activity measures: median (IQR)**					
SLEDAI-2K	2(0–4)	2 (0–4)	2 (2–4)	2 (0–2)	2 (2–4)
PGA	0 (0–1)	0 (0–1)	0 (0–0)	0 (0–1)	0 (0–1)
Patient-reported NRS	7 (6–8)	8 (7–8)	8 (7–9)	7 (7–8)	8 (7–9)
**Serology: n (%)**					
Anti-dsDNA	87 (65)	56 (58)	18 (47)	5 (38)	10 (43)
Low complement (C3 and/or C4)	71 (52)	43 (44)	16 (42)	4 (31)	13 (57)
**Treatment status: n (%)**					
Hydroxychloroquine	120 (90)	88 (92)	32 (84)	11 (85)	19 (83)
Immunosuppressants					
MTX	12 (9)	7 (7)	1 (2)	0 (0)	1 (4)
AZA	24 (18)	18 (18)	7 (18)	2 (15)	5 (22)
MMF	44 (32)	38 (39)	7 (18)	2 (15)	5 (22)
CyA	4(3)	3 (3)	1 (3)	0 (0)	1 (4)
Belimumab	21 (15)	16 (16)	3 (8)	1 (8)	2 (9)
**Treatment changes: n (%)**					
Corticosteroid tapering	21 (16)	10 (10)	**11 (29)** *	4 (31)	6 (26)
Corticosteroid discontinuation	13 (10)	9 (9)	4 (10)	2 (5)	2 (9)
Immunosuppressant discontinuation	0 (0)	0 (0)	0 (0)	0 (0)	0 (0)

SLEDAI-2K: Systemic Lupus Erythematosus Disease Activity Index 2000; PGA: physician global assessment scale; NRS: numerical rating scale; MTX: methotrexate; AZA: Azathioprine; MMF: mycophenolate mofetil; CyA: cyclosporine A. *: *p* < 0.05 compared to OFs.

**Table 3:** Clinical and laboratory features at time of flare.

	All Flares (n = 134)	OFs (n = 96)	IAFs (n = 38)	Viral IAFs (n = 13)	Bacterial IAFs (n = 23)
**Disease activity measures: median (IQR)**					
SLEDAI-2K	5 (4–6)	5 (4–6)	5 (3–6)	3 (0–6)	6 (4–6)
Delta SLEDAI-2K	2 (0–4)	2 (0–4)	2 (0–5)	1 (0–4)	4 (2–5)
Total BILAG score	1 (1–2)	1 (0–1)	1 (1–2)	1 (0–2)	2 (1–2)
PGA	1 (0–1)	1 (0–1)	1 (0–1)	1 (0–1)	1 (1–1)
Patient-reported NRS	7 (6–8)	7 (6–8)	7 (5–8)	6 (4–7)	7 (5–8)
**Serology: n(%)**					
Anti-dsDNA	87 (65)	64 (67)	23 (61)	**5 (38)** *	16 (74)
Low complement (C3 and/or C4)	71 (53)	53 (55)	18 (47)	3 (23)	12 (57)
**Other laboratory features: median (IQR)**					
Hb (g/dL)	12.8 (12–14)	1.8 (11.8–14)	12.6 (11.6–13.7)	13 (11–13)	12.5 (12–14)
Platelets × 10^3^/microlitre	227 (182–269)	239(189–277)	210 (169–262)	177 (162–236) *	215 (177–262)
WBCs/microlitre	5000 (3600–6675)	5375 (3875–6825)	4510 (3100–5547)	4630 (2880–6720)	4400 (3100–5050)
Neutrophils (%)	61 (53–67)	61 (55–66)	60 (47–68)	62 (49–71)	59 (42–66)
Lymphocytes (%)	26 (53–67)	26 (19–32)	27 (19–40)	25 (18–35)	29 (20–41)
Monocytes (%)	9 (7–12)	9 (7–12)	10 (8–12)	9 (8–11)	11 (9–12)
Eosinophils (%)	2 (1–3)	2 (1–3)	2 (1–3)	2 (1–3)	3 (1–4)
Basophils (%)	1 (0–1)	0 (0–1)	1 (0–1)	1 (0–1)	1 (0–1)
Serum creatinine (mg/dL)	0.8 (0.6–0.9)	0.8 (0.6–0.9)	0.8 (0.6–1)	0.8 (0.8–1.1)	0.7 (0.7–1)
AST (U/L)	22 (17–26)	21 (17–25)	23 (17–27)	19 (13–28)	24 (20–29)
ALT (U/L)	17 (13–23)	17 (13–22)	19 (14–29)	19 (15–31)	20 (14–28)

SLEDAI-2K: Systemic Lupus Erythematosus Disease Activity Index 2000; BILAG: British Isles Lupus Assessment Group; PGA: physician global assessment scale; NRS: numerical rating scale; Hb: haemoglobin; WBCs: white blood cells; ALT: alanine aminotransferase; AST: aspartate aminotransferase. *: *p* < 0.05 compared to OFs.

## Data Availability

Data supporting this research can be shared upon reasonable request to the corresponding author.

## Supplementary Materials

The following supporting information can be downloaded at: https://www.mdpi.com/article/10.3390/pathogens13110934/s1, Table S1: LLDAS retention over time.
