# Supplementary material for: Infection-Associated Flares in Systemic Lupus Erythematosus
Source: Pathogens. 2024 Oct 26;13(11):934. doi: 10.3390/pathogens13110934 (PMC11597141; doi:10.3390/pathogens13110934)
Supplement: Supplementary file 1 [file pathogens-13-00934-s001.zip › Supplemental Table S1.pdf]

Supplemental Table S1: LLDAS retention over time

	Year 1	Year 2	Year 3	Year 4	Year 5	>5 years
**Patients with flares (annual): n**	60	22	16	11	2	3
**Patients with flares (cumulative): n**	60	82	98	109	111	114
**Patients at risk (annual): n**	242	150	99	54	27	25
**Patients at risk (cumulative): n**	242	210	181	152	136	136
**LLDAS loss rate (annual): %**	25%	15%	16%	7%	12%	12%
**LLDAS loss rate (cumulative): %**	25%	39%	54%	72%	82%	84%
**LLDAS retention rate (annual): %**	75%	85%	84%	93%	88%	88%
**LLDAS retention rate (cumuative): %**	75%	61%	46%	28%	18%	16%
